# Comparison of manual and automatic MR‐CT registration for radiotherapy of prostate cancer

**DOI:** 10.1120/jacmp.v17i3.6088

**Published:** 2016-05-08

**Authors:** Anne Sofie Korsager, Jesper Carl, Lasse Riis Østergaard

**Affiliations:** ^1^ Department of Health Science and Technology Aalborg University Denmark; ^2^ Department of Medical Physics Oncology, Aalborg University Hospital Denmark

**Keywords:** image registration, magnetic resonance, computed tomography, radiation therapy, prostate cancer

## Abstract

In image‐guided radiotherapy (IGRT) of prostate cancer, delineation of the clinical target volume (CTV) often relies on magnetic resonance (MR) because of its good soft‐tissue visualization. Registration of MR and computed tomography (CT) is required in order to add this accurate delineation to the dose planning CT. An automatic approach for local MR‐CT registration of the prostate has previously been developed using a voxel property‐based registration as an alternative to a manual landmark‐based registration. The aim of this study is to compare the two registration approaches and to investigate the clinical potential for replacing the manual registration with the automatic registration. Registrations and analysis were performed for 30 prostate cancer patients treated with IGRT using a Ni‐Ti prostate stent as a fiducial marker. The comparison included computing translational and rotational differences between the approaches, visual inspection, and computing the overlap of the CTV. The computed mean translational difference was 1.65, 1.60, and 1.80 mm and the computed mean rotational difference was 1.51°, 3.93°, and 2.09° in the superior/inferior, anterior/posterior, and medial/lateral direction, respectively. The sensitivity of overlap was 87%. The results demonstrate that the automatic registration approach performs registrations comparable to the manual registration.

PACS number(s): 87.57.nj, 87.61.‐c, 87.57.Q‐, 87.56.J‐

## I. INTRODUCTION

Planning image‐guided radiotherapy (IGRT) of prostate cancer often benefits from several imaging technologies. Computed tomography (CT) is the standard imaging modality for planning of IGRT of prostate cancer. However, studies have shown that a delineation of the clinical target volume (CTV) using CT leads to an overestimation of the CTV. CTVs up to 40% larger have been found on CT when compared to using magnetic resonance (MR) for CTV delineation.^1^, ^2^, ^3^ As a consequence, MR imaging is commonly used for accurate soft‐tissue delineation of the prostate as the CTV and CT images are used for radiation dose calculations. The use of multimodal imaging requires registration of MR and CT for mapping accurate soft‐tissue delineations from MR to CT for dose planning.

An important challenge in the registration process relates to the movement of the prostate between scans, which can occur relatively to the pelvic bones and neighboring organs in the pelvic. This may result in an inaccurate alignment of the prostate in MR and CT if the registration is based on an alignment of the pelvic bones.^4^, ^5^, ^6^, ^7^ The movement can be accounted for in a conservative planning based on alignment of pelvic bones or external contours and adding large margins to the CTV. However, this may result in increased radiation toxicity of the normal surrounding tissue, such as the rectum and bladder.^(8)^

To be able to reduce margins added to the CTV, a local registration of the prostate is needed to ensure an accurate alignment of the prostate in MR and CT. A local registration can be obtained by fiducial markers, which are implanted into the prostate gland and can be regarded as common visualized structures in MR and CT. Previous studies have performed local rigid MR‐CT registration of the prostate based on registrations using implanted fiducial markers. Landmark‐based registration,^8^, ^9^, ^10^ segmentation‐based registration,^(11)^ or voxel property‐based registration^12^, ^13^, ^14^ have all been applied. Automatic image registration benefits from objectivity in the registrations as the registration quality is observer‐independent. Furthermore, it saves manual labor in the clinic as the observer does not need to select corresponding landmarks in MR and CT.

The use of fiducial markers in a rigid registration of MR and CT, as in the previously listed studies, relies on an assumption that the prostate does not deform but only moves relatively to the pelvic bones. This assumption has been investigated and discussed in a number of studies.^15^, ^16^, ^17^, ^18^, ^19^ Kerkhof et al.^(15)^ investigated the prostate deformation based on manual prostate delineations in MR scans. They found that prostate deformation can occur due to differences in rectum filling, but that it was difficult to distinguish the delineation uncertainties and the prostate deformation. The conclusion of the study was that with the currently used margins of more than 4 mm and the treatment divided into fractions, the increase of rectum dose caused by prostate deformation can be negligible. These findings are supported in the studies by van der Wielen et al.,^(16)^ Dehnad et al.,^(20)^ and Deurloo et al.^(17)^ where fiducial markers implanted into the prostate and CT prostate delineation, respectively, were used to investigate the prostate deformation. Other studies^18^, ^19^ have found that prostate deformation can occur and is an important source of uncertainty in radiotherapy of prostate cancer. An endorectal coil is frequently used to improve the MR quality and to limit prostate movement. However, the use of an endorectal coil causes deformations of the prostate,^(21)^ which complicates the registration process by requiring a need for a nonrigid registration. Limitations in studies investigating prostate deformation include a possible migration of the fiducial markers in cases where they are used as surrogates for deformation, as was done in the van der Wielen^(16)^ and Deurloo studies,^(17)^ and delineation errors when prostate delineations are used as surrogates for prostate deformation, as in the work by Kerhof et al.,^(15)^ and Deurloo et al.^(17)^

Comparison of image registration approaches have previously been performed in a number of studies.^22^, ^23^, ^24^, ^25^, ^26^ West et al.^(22)^ established a ground‐truth registration using a manual landmark‐based registration, which can be used for comparison of new approaches for brain registration. Volume overlap measures in form of Jaccard index or Dice similarity coefficient (DSC)^24^, ^25^, ^26^ and distance measures in form of Hausdorff distance and mean surface distance^25^, ^26^ have been used to compare registration methods.

Carl et al.^10^, ^27^ have presented a removable thermoexpandable Ni‐Ti prostate stent as an alternative to gold markers, which is now used in the clinic at Aalborg University Hospital. A recent study has found a significantly smaller CTV and reduced urinary frequency and urinary retention toxicity scores using MR images followed by a MR‐CT registration using the stent as a fiducial marker, when compared to long‐term toxicity when CT was used for prostate delineation.^(28)^

The objective of this paper is to compare two approaches previously presented for MR‐CT registration that have used the Ni‐Ti prostate stent as the fiducial marker. The first approach is based on manually defined landmarks on the prostate stent and on the pelvic bones and is now the clinical practice at Aalborg University Hospital.^(10)^ The second approach is an automatic approach using the voxel similarity measure mutual information for a local registration of the prostate stent and a tightly surrounding volume.^(14)^ The comparison was performed by comparing the translational and rotational differences for the CTV volume and anatomical overlap of the CTV between the two approaches.

## II. MATERIALS AND METHODS

### A. Imaging data acquisition

Data from 30 patients was used and consisted of spiral CT images (GE Medical Systems, San Francisco, CA) with an image matrix of 512×512×16 corresponding to a voxel size of $1\times 1\times 2.5\;{\text{mm}}^{3}$ (2.5 mm slice thickness, 2.5 spacing, 120 kVp, acquisition time 20 s) and axial $T_{2}-\text{weighted}$ (FSE, TE = 81 ms, TR = 2800 ms, NA = 2, 3 mm slice thickness, 3 mm spacing, acquisition time 15 min) 1.5 Tesla MR scans (GE Medical Systems) obtained with a body coil with an image matrix of 512×512×16 corresponding to a voxel size of $0.55\times 0.55\times 3\;{\text{mm}}^{3}$. Only $T_{2}-\text{weighted}$ MR images were acquired. Both axial MR and axial CT scans were acquired supine using knee and feet flexion, and the time between the two scans was between zero and five days. No special measures were taken to reduce motion.

The patients were all diagnosed with local or locally advanced prostate cancer and referred to the Department of Oncology at Aalborg University Hospital for radiotherapy treatment. The prostate was manually delineated by an experienced clinician slice‐wise on the axial MR and was used as the CTV for the radiotherapy treatment. The insertion of the prostate stent was performed using locally urethral anesthesia and prophylactic antibiotics (Ibrufen and Ciproxin). The catheter was inserted to the correct position and the collar of the stent was then expanded using 50 ml 60°C hot water. The length of the stent was adapted to the individual patient based on an estimation of the length of the urethral part of prostate using a diagnostic MR scan. The outer diameter of the cylinder shape part of the stent was 7 mm and for the fully expanded collar 14 mm. The noninvasive insertion procedure, together with a possibility of easy removal of the prostate stent, is one of the benefits of the prostate stent compared with alternative fiducial markers. Advantages using the prostate stent in the MR‐CT registration include fewer artifacts in CT and the fact that it is clearly visible in MR. Other advantages are that the stent itself is one large 3D object and it does not migrate over time.^10^, ^29^

### B. Methods for MR‐CT registration

The manual approach used four or five selected anatomical landmarks to perform a rigid registration of MR and CT. Three landmarks were defined on the prostate stent, one each at the cranial, middle, and caudal end of the stent. Another one or two anatomical landmarks were defined, one anterior and one lateral to the prostate surface but as close to the prostate surface as possible. To weight the landmarks on the stent as much as possible, it was sought only to use one anatomical landmark placed at the symphysis pubis between the two pubic arches. In the cases with large prostate movement, another landmark was placed in one of the pelvic bones. The anatomical landmarks were moved if they had an impact of the alignment of the prostate stent in MR and CT. The anatomical landmarks were defined to constrain rotations about the central axis of the stent. The registration that resulted in a minimized root‐mean‐square distance between landmarks was then determined and the MR image was transformed. Manual registrations were performed between March 2007 and May 2009^(10)^ using Eclipse (Varian Medical Systems) in which the root‐mean‐square distance between each landmark pair was automatically minimized.

The automatic approach performed a rigid registration in a two‐step voxel property‐based approach, which used the voxel similarity measure mutual information presented by Collignon et al.^(30)^ in both steps. The goal of the first step was to align the pelvic bones, which was achieved in the first registration step using all voxels in the image datasets. The first step was used as an initial alignment and to enable rotational constraints to prevent rotations about the central axis of the stent. Second, a local registration using the rotational constraints was performed based on a manually defined region, which tightly surrounded the prostate stent.^(14)^ The registration was performed using the registration tool minctracc (MINC Tool Kit, McConnell Brain Imaging Centre, Montreal, Canada).^(31)^

### C. Methods for comparison of registrations

Three experiments were performed to compare the registration approaches.

The first experiment investigated translational and rotational differences of the CTV after registration with each of the two registration approaches. This was obtained by extracting 1000 random point coordinates automatically inside the CTV volume in MR to investigate the difference in translation and rotation of tumor, which was included in the CTV. The 1000 points were then registered with each of the two registration approaches, and the rotational and translational differences after registration were calculated, as well as the distance between the random points after registration.

The second experiment investigated the registration effect on the CTV by computing the overlap of CTVs produced by each registration. Identical transformations will give an overlap of one and the larger the differences in the transformations, the lower the overlap. The overlap was calculated as the DSC (Eq. (1)) and sensitivity (Eq. (2)).

(1) $$DCS(CTV_{man'}CTV_{auto})=\frac{2\left|CTV_{man}\cap CTV_{auto}\right|}{\left|CTV_{man}+CTV_{auto}\right|},$$

where ${CTV}_{man}$ is the CTV after the manual registration and ${CTV}_{auto}$ is the CTV after the automatic registration.

(2) $$Sensitivity=\frac{Number\;of\;common\;voxels}{Number\;of\;common\;voxels+Number\;of\;uncommon\;voxels}$$

Sensitivity was used as a measure of the number of common voxels in the CTV between the two registration approaches compared to the number of uncommon voxels in CTV between the registration approaches. Furthermore, the prostate stent and the pelvic bones were segmented using fuzzy c‐means to visualize the differences in transformations on the prostate stent with reference to the pelvic bones.

The third experiment investigated the potential for the automatic registration approach to be implemented into clinical practice. This was indirectly validated by extending the manual delineation of the CTV after the automatic registration with the error of the automatic registration. The metrics calculated in the volume overlap experiment were calculated in this experiment, as well. The overlap between the volumes were expected to be higher as the CTV transformed with the automatic registration approach was extended, which was expected also to result in a higher sensitivity.

## III. RESULTS

### A. Comparison of translational and rotational differences

**Figure 1:**
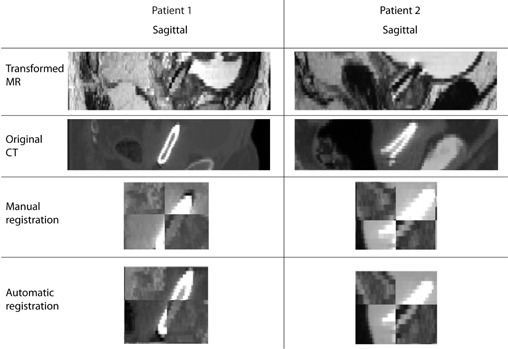
Sagittal view of the transformed MR, original CT, checkerboard representation of the registration result of the manual registration, and checkerboard representation of the registration result of the automatic registration for two patients.

Figure 1 shows two representative examples of the registration results obtained from the two registration approaches. As seen in the checkerboard representation of the results, similar results of the prostate stent alignment in MR and CT were obtained. Furthermore, both registration approaches obtained an accurate alignment of the prostate stent in transformed MR and CT.

Table 1 shows the translational and rotational difference for the CTV registered with each of the two registration approaches. The translational difference was determined by the distance between the corresponding points from the CTV after registration with each registration approach.

The rotational difference in the z‐axis (superior/inferior direction) can be explained by the rotational constraints in the automatic registration approach. In this approach, no rotation about the z‐axis was allowed to prevent rotation about the central axis of the stent.

**Table 1:** The registration translational difference $T_{D}$ and rotational difference $R_{D}$ calculated as the absolute difference in image plane between 1000 randomly selected points from the CTV. The mean, standard deviation (SD), and range of the results are shown.

	$T_{D}\;mean\pm SD$ (range) (mm)	$R_{D}\;mean\pm SD$ *range (deg)*
Superior/inferior	1.82±1.50 (0.02–9.57)	1.51±1.36 (0.01–5.12)
Anterior/posterior	1.66±1.01 (0.01–4.61)	3.93±3.79 (0.00–14.19)
Medial/lateral	1.93±1.24 (0.00–7.25)	2.09±1.98 (0.01–7.71)

### B. Anatomical overlap

The CTV overlap after registrations measured as the mean DSC was 0.87±0.05 (range 0.77‐0.95) and the mean sensitivity was 0.87±0.05 (range 0.74‐0.95).

**Figure 2:**
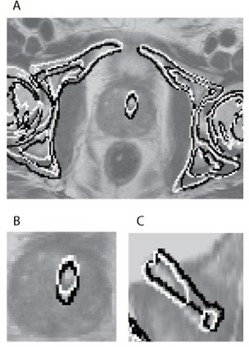
The two contours are obtained from a bone and prostate stent segmentation in CT followed by an inverse transformation of the registration by the automatic (white) and the manual (black) registration for one patient. The segmentations are superimposed on the original $T_{2}-\text{weighted}$ MR images: (a) bones and prostate stent segmentation in the axial plane; (b) region of interest and the prostate stent in the axial plane; (c) region of interest and the prostate stent in the sagittal plane. It can be noticed, in particular in image (a), that both methods are based on a registration of the prostate stent and a tightly surrounding volume as either the black or white contour follow the contour of the pelvic bone. This results in an accurate alignment of the prostate stent in image (b), which indicates that the prostate has moved relatively to the pelvic bones. In image (c), a gap in the segmentation of the prostate stent can be observed from the automatic registration caused by interpolation.

Figure 2 shows the bone and prostate stent segmentations superimposed on the original MR image to visualize the translations and rotations of the pelvic bones and prostate stent.

### C. Potential of using automatic registration

The mean registration error calculated as the Euclidean distance for the landmark‐based MR‐CT registration used in the present study has previously been calculated and can be regarded as the general registration error. The mean error was 1.25 mm and the maximum error was 1.86 mm between the four or five anatomical landmarks.^(10)^ The registration error for the automatic registration is more difficult to determine, but was measured in a previous phantom study to be 0.97 mm computed as a root‐mean‐square error.^(14)^ This error was extended with a 95% confidence interval to determine the margin, which was added to the CTV. The margin determined by the registration error of the automatic registration was added to the CTV after automatic registration. The overlap was then compared with the original CTV after manual registration.

**Figure 3:**
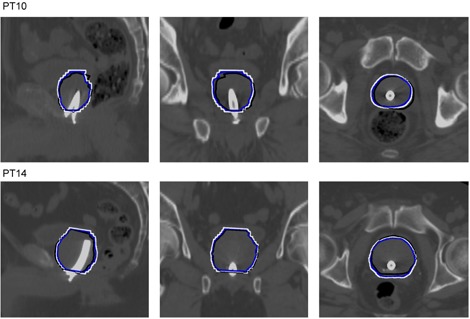
The order is sagittal, coronal, and axial views of the overlap of the manual prostate delineation performed in the original MR image and then aligned to the CT based on the manual registration (black), the automatic registration of the CTV (blue), and the automatic registration of the extended CTV (white). The delineations are superimposed on the CT images.

Figure 3 shows the contour overlap of the registration approaches from the dataset with the largest and the dataset with the smallest difference between the anatomical overlap after registration. The white contour is the extended CTV obtained from the blue contour. It can be seen that there was a larger overlap between the CTV after the manual registration and with the extended CTV.

The mean sensitivity between the manual delineation after manual registration and the extended manual delineation after automatic registration was 0.90±0.05 (range 0.77‐0.97). The mean DSC was 0.87±0.05 (range 0.77‐0.93). The sensitivity increased as both the rate of true positive voxels increased and the rate of false negative voxels decreased.

## IV. DISCUSSION

Several registration approaches have been published and are used for registration of MR and CT images of the prostate. In this paper, a comparison of two of the most widely used approaches, landmark‐based registration using manually defined landmarks and voxel property‐based registration using mutual information as the similarity measure, is presented. The registrations from the manual landmark‐based registration were obtained from the treatment planning process and were, therefore, not performed by the authors, to eliminate any potential observer bias.

The comparison was based on differences in the translations and rotations between the two registration approaches. Also, a manual delineation of the prostate as the CTV for each dataset was used to calculate the anatomical overlap of the prostate in the transformed MR images. Both registration approaches assume no prostate deformation or change in prostate volume caused by differences in rectum or bladder filling between the scans or the use of an endorectal coil. This assumption is supported by studies where the prostate deformation was found to be present, but negligible, compared with the prostate movement relative to the pelvic bones.^15^, ^16^, ^17^ A rigid registration will in cases where prostate deformations are present for example when using an endorectal coil result in a reduced coverage of the prostate volume during radiotherapy treatment. The partial volume effect makes the prostate stent appear larger in CT with an increased diameter of less than 1 mm compared with MR. The partial volume effect was addressed in the automatic registration as all computations were performed using trilinear interpolations and in the world coordinate system instead of voxel coordinate system.

Otherwise, the registrations would have introduced a risk that the prostate stent in MR was aligned with one of the stent border in CT instead of an alignment of the central axis in both imaging modalities. No problems caused by partial volume effect could be found in either of the registration approaches. Artifacts are a common problem in CT when fiducial markers are used. The prostate stent in the present study is made of nickel and titanium, which both have a low atomic number. Therefore, the artifacts caused by the prostate stent in CT are less of a problem compared to gold fiducial markers.

The processing time of the automatic registration was in total between 3 and 4 min for each patient. This was merely machine processing time and included saving all intermediate results and without speed improvements. For the manual registration, the time required for a clinician to perform the registration will vary, but typically takes between 10 and 30 min and is entirely manual labor except for the minimization of the distance between the landmarks.

No ground‐truth registration exists, which makes visual inspection crucial in order to assess the influence of the difference between the registration approaches. It is possible to perform phantom studies to get closer to the ground truth, as in Korsager et al.^(14)^ Other studies that have compared registrations in brain imaging (e.g. West et al.^(22)^) have used landmarks defined on implanted fiducial markers after registrations to calculate the difference in transformations (fiducial registration error), and Sarkar et al.^(23)^ used manually defined anatomical landmarks. Studies performing MR‐CT registration often define the manual registration as the ground truth and then validate the accuracy of the alternative method based on the differences from the manual registration.^(11)^ However, a manual registration is also prone to errors as it depends on the observers ability to select corresponding landmarks in MR and CT.^(11)^ The interobserver variation of the landmark‐based registrations used in this study is unknown but it has previously been studied. Kitamura et al.^(32)^ studied the interobserver variation in the selection of the center of mass of fiducial markers implanted into the prostate gland in CT images. The standard deviation of the variation was 0.4 mm, however, only CT images were used and the variation of marker selection can be assumed to be higher in MR as the fiducial markers are less well defined in MR. De Brabandere et al.^(33)^ compared interobserver variability and uncertainties in seed definition in CT and MRI used for planning prostate brachytherapy. The interobserver variability in seed definition was 1.1±0.5 mm for CT and 3.0±0.9 mm for MR.^(33)^ Huisman et al.^(11)^ compared a manual landmark‐based registration with an iterative closest point registration and found that the quality of the landmark‐based registration was observer‐independent. An automatic registration is not observer‐dependent, however, there is a risk that the registration will be trapped in a local extremum or will be anatomically unrealistic. This stresses the importance of visual inspection. The clinical potential of the automatic registration approach was indirectly validated by adding a margin corresponding to the automatic registration error to the CTV and comparing it to the original CTV and manual registration. The validation showed a general good coherence between the registration approaches. The potential also needs to be compared with the uncertainties related to the manual registration.

The error which is used to discuss the clinical potential is most likely an overestimate of the automatic registration error as it is computed as a root‐mean‐square error and thereby a worst‐case estimate, assuming an isotropic error in all three image directions. Furthermore, it is a systematic error determined from one patient. A more appropriate procedure is to find the mean systematic and mean random error for a number of patients and use that to compute the error, which needs to the accounted for in the margin added to the CTV. As the validations have not been performed using the same procedure and datasets, a comparison of the accuracy cannot be performed.

The errors in the registration and the difference between the registration approaches need to be accounted for in radiotherapy planning. This is done by adding a margin to the CTV, which also includes uncertainties related to patient setup errors and prostate position error caused by interfraction movement and by intrafraction movement. However, the benefit of using image registration in prostate cancer radiotherapy is that it enables target delineation in MR, which has been shown to reduce the delineated volume by about 30%–40%^1^, ^2^, ^3^ and has the potential to reduce long‐term toxicity.^(28)^ The interfraction movement and intrafraction movement of the prostate can be reduced if the prostate is tracked continuously during all treatments. It is possible using X‐ray imaging to monitor the prostate stent and either pause the radiotherapy treatment or to reposition the patient in the treatment system. This will likely enable a reduction of the margin added to the CTV from the currently used of 5 mm to approximately 3‐4 mm. However, there is not much that suggests that it will provide a reduction in the side effects of the treatments with the currently used radiation doses.^34^, ^35^ The risk of recurrence has been shown to increase if the margin added to CTV is to small.^(36)^ These conditions might change if higher radiation doses are introduced.

In the present study, only $T_{2}-\text{weighted}$ MR scans were available as they are now used in the clinic to delineate the prostate as the CTV. Other MR modalities, such as Dynamic Contrast Enhanced MRI and Diffusion‐weighted MRI, would be appropriate to include to define the tumor and thereby increase confidence in prostate tumor definition.

## V. CONCLUSIONS

The results demonstrate comparable registrations between the manual landmark‐based registration and the automatic voxel property‐based registration. The quality of the automatic MR‐CT registration is observer‐independent, which improves objectivity and reduces uncertainties in the planning.

## COPYRIGHT

This work is licensed under a Creative Commons Attribution 4.0 International License.

## References

[1] Gao Z , Wilkins D , Eapen L , Morash C , Wassef Y , Gerig L . A study of prostate delineation referenced against a gold standard created from the visible human data. Radiother Oncol. 2007;85(2):239–46. 17825447 10.1016/j.radonc.2007.08.001

[2] Hentschel B , Oehler W , Strauβ D , Ulrich A , Malich A . Definition of the CTV prostate in CT and MRI by using CT‐MRI image fusion in IMRT planning for prostate cancer. Strahlenther Onkol. 2011;187(3):183–90. 21347638 10.1007/s00066-010-2179-1

[3] Rasch C , Barillot I , Remeijer P , van Herk M , Lebesque JV . Definition of the prostate in CT and MRI: a multiobserver study. Int J Radiat Oncol Biol Phys. 1999;43(1):57–66. 9989514 10.1016/s0360-3016(98)00351-4

[4] Langen KM and Jones DTL . Organ motion and its management. Int J Radiat Oncol Biol Phys. 2001;50(1):265–78. 11316572 10.1016/s0360-3016(01)01453-5

[5] Shang Q , Sheplan Olsen LJ , Stephans K , Tendulkar R , Xia P . Prostate rotation detected from implanted markers can affect dose coverage and cannot be simply dismissed. J Appl Clin Med Phys. 2013;14(3):4262. 23652257 10.1120/jacmp.v14i3.4262 PMC5714427

[6] Schallenkamp JM , Herman MG , Kruse JJ , Pisansky TM . Prostate position relative to pelvic bony anatomy based on intraprostatic gold markers and electronic portal imaging. Int J Radiat Oncol Biol Phys. 2005;63(3):800–11. 16199313 10.1016/j.ijrobp.2005.02.022

[7] Hoogeman MS , van Herk M , de Bois J , Lebesque JV . Strategies to reduce the systematic error due to tumor and rectum motion in radiotherapy of prostate cancer. Radiother Oncol. 2005;74(2):177–85. 15734206 10.1016/j.radonc.2004.12.010

[8] Parker CC , Damyanovich A , Haycocks T , Haider M , Bayley A , Catton CN . Magnetic resonance imaging in the radiation treatment planning of localized prostate cancer using intra‐prostatic fiducial markers for computed tomography co‐registration. Radiother Oncol. 2003;66(2):217–24. 12648794 10.1016/s0167-8140(02)00407-3

[9] Crook J , McLean M , Yeung I , Williams T , Lockwood G . MRI‐CT fusion to assess postbrachytherapy prostate volume and the effects of prolonged edema on dosimetry following transperineal interstitial permanent prostate brachytherapy. Brachytherapy. 2004;3(2):55–60. 15374536 10.1016/j.brachy.2004.05.001

[10] Carl J , Nielsen J , Holmberg M , Højkjaer Larsen E , Fabrin K , Fisker RV . A new fiducial marker for Image‐guided radiotherapy of prostate cancer: clinical experience. Acta Oncol (Madr). 2008;47(7):1358–66. 10.1080/02841860802241972 18618341

[11] Huisman HJ , Fütterer JJ , van Lin ENJT , et al. Prostate cancer: precision of integrating functional MR imaging with radiation therapy treatment by using fiducial gold markers. Radiology. 2005;236(1):311–17. 15983070 10.1148/radiol.2361040560

[12] Vidakovic S , Jans HS , Alexander A , Sloboda RS . Post‐implant computed tomography‐magnetic resonance prostate image registration using feature line parallelization and normalized mutual information. J Appl Clin Med Phys. 2006;8(1):21–32. 17592452 10.1120/jacmp.v8i1.2351 PMC5722399

[13] Roberson PL , McLaughlin PW , Narayana V , Troyer S , Hixson GV , Kessler ML . Use and uncertainties of mutual information for computed tomography/magnetic resonance (CT/MR) registration post permanent implant of the prostate. Med Phys. 2005;32(2):473–82. 15789594 10.1118/1.1851920

[14] Korsager AS , Carl J , Østergaard LR . MR‐CT registration using a Ni‐Ti prostate stent in image‐guided radiotherapy of prostate cancer. Med Phys. 2013;40(6):061907. 23718598 10.1118/1.4807087

[15] Kerkhof EM , van der Put RW , Raaymakers BW , van der Heide UA , van Vulpen M , Lagendijk JJ . Variation in target and rectum dose due to prostate deformation: an assessment by repeated MR imaging and treatment planning. Phys Med Biol. 2008;53(20):5623–34. 18799831 10.1088/0031-9155/53/20/004

[16] van der Wielen GJ , Mutanga TF , Incrocci L , et al. Deformation of prostate and seminal vesicles relative to intraprostatic fiducial markers. Int J Radiat Oncol Biol Phys. 2008;72(5):1604–11. 19028284 10.1016/j.ijrobp.2008.07.023

[17] Deurloo KE , Steenbakkers RJ , Zijp LJ , et al. Quantification of shape variation of prostate and seminal vesicles during external beam radiotherapy. Int J Radiat Oncol Biol Phys. 2005;61(1):228–38. 15629616 10.1016/j.ijrobp.2004.09.023

[18] Mayyas E , Kim J , Kumar S , et al. A novel approach for evaluation of prostate deformation and associated dosimetric implications in IGRT of the prostate. Med Phys. 2014;41(9):091709. 25186384 10.1118/1.4893196

[19] Mutanga TF , de Boer HCJ , van der Wielen GJ , Hoogeman MS , Incrocci L , Heijmen BJ . Margin evaluation in the presence of deformation, rotation, and translation in prostate and entire seminal vesicle irradiation with daily marker‐based setup corrections. Int J Radiat Oncol Biol Phys. 2011;81(4):1160–67. 21035957 10.1016/j.ijrobp.2010.09.013

[20] Dehnad H , Nederveen AJ , van der Heide UA , van Moorselaar RJ , Hogman P , Lagendijk JJ . Clinical feasibility study for the use of implanted gold seeds in the prostate as reliable positioning markers during megavoltage irradiation. Radiother Oncol. 2003;67(3):295–302. 12865177 10.1016/s0167-8140(03)00078-1

[21] Kim Y , Hsu I‐CJ , Pouliot J , Noworolski SM , Vigneron DB , Kurhanewicz J . Expandable and rigid endorectal coils for prostate MRI: impact on prostate distortion and rigid image registration. Med Phys. 2005;32(12):3569–78. 16475755 10.1118/1.2122467

[22] West J , Fitzpatrick JM , Wang MY , et al. Comparison and evaluation of retrospective intermodality brain image registration techniques. J Comput Assist Tomogr. 1997;21(4):554–66. 9216759 10.1097/00004728-199707000-00007

[23] Sarkar A , Santiago RJ , Smith R , Kassaee A . Comparison of manual vs. automated multimodality (CT‐MRI) image registration for brain tumors. Med Dosim. 2005;30(1):20–24. 15749007 10.1016/j.meddos.2004.10.004

[24] Castadot P , Lee JA , Parraga A , Geets X , Macq B , Grégoire V . Comparison of 12 deformable registration strategies in adaptive radiation therapy for the treatment of head and neck tumors. Radiother Oncol. 2008;89(1):1–12. 18501456 10.1016/j.radonc.2008.04.010

[25] Pantazis D , Joshi A , Jiang J , et al. Comparison of landmark‐based and automatic methods for cortical surface registration. Neuroimage. 2010;49(3):2479–93. 19796696 10.1016/j.neuroimage.2009.09.027 PMC2818237

[26] Varadhan R , Karangelis G , Krishnan K , Hui S . A framework for deformable image registration validation in radiotherapy clinical applications. J Appl Clin Med Phys. 2013;14(1):4066. 23318394 10.1120/jacmp.v14i1.4066 PMC3732001

[27] Carl J , Nielsen J , Holmberg M , Larsen EH , Fabrin K , Fisker RV . Clinical results from first use of prostate stent as fiducial for radiotherapy of prostate cancer. Acta Oncol. 2011;50(4):547–54. 21174520 10.3109/0284186X.2010.541935

[28] Sander L , Langkilde NC , Holmberg M , Carl J . MRI target delineation may reduce long‐term toxicity after prostate radiotherapy. Acta Oncol. 2014;53(6):809–14. 24358954 10.3109/0284186X.2013.865077

[29] Carl J , Lund B , Larsen EH , Nielsen J . Feasibility study using a Ni‐Ti stent and electronic portal imaging to localize the prostate during radiotherapy. Radiother Oncol. 2006;78(2):199–206. 16413623 10.1016/j.radonc.2005.11.015

[30] Collignon A , Maes F , Delaere D , Vandermeulon D , Suetens P , Marchal G . Automated multi‐modality image registration based on information theory. In: Bizais Y, Barillot C, Di Paola R, editors. Information processing in medical imaging. Springer Netherlands: 1995 p. 264–74.

[31] Collins DL , Neelin P , Peters TM , Evans AC . Automatic 3D intersubject registration of MR volumetric data in standardized Talairach space. J Comput Assist Tomogr. 1994;18(2):192–205. 8126267

[32] Kitamura K , Shirato H , Shimizu S , et al. Registration accuracy and possible migration of internal fiducial gold marker implanted in prostate and liver treated with real‐time tumor‐tracking radiation therapy (RTRT). Radiother Oncol. 2002;62(3):275–81. 12175558 10.1016/s0167-8140(02)00017-8

[33] De Brabandere M , Al‐Qaisieh B , De Wever L , et al. CT‐ and MRI‐based seed localization in postimplant evaluation after prostate brachytherapy. Brachytherapy. 2013;12(6):580–88. 23876358 10.1016/j.brachy.2013.06.003

[34] Bruner DW , Hunt D , Michalski JM , et al. Preliminary patient‐reported outcomes analysis of 3‐dimensional radiation therapy versus intensity‐modulated radiation therapy on the high‐dose arm of the Radiation Therapy Oncology Group (RTOG) 0126 prostate cancer trial. Cancer. 2015;121(14):2422–30. 25847819 10.1002/cncr.29362 PMC4490066

[35] Crehange G , Mirjolet C , Gauthier M , et al. Clinical impact of margin reduction on late toxicity and short‐term biochemical control for patients treated with daily on‐line image guided IMRT for prostate cancer. Radiother Oncol. 2012;103(2):244–46. 22119374 10.1016/j.radonc.2011.10.025

[36] Engels B , Soete G , Verellen D , Storme G . Conformal arc radiotherapy for prostate cancer: increased biochemical failure in patients with distended rectum on the planning computed tomogram despite image guidance by implanted markers. Int J Radiat Oncol Biol Phys. 2009;74(2):388–91. 19056185 10.1016/j.ijrobp.2008.08.007

